# The Threshold Effect of Knowledge Diversity on Urban Green Innovation Efficiency Using the Yangtze River Delta Region as an Example

**DOI:** 10.3390/ijerph191710600

**Published:** 2022-08-25

**Authors:** Han Bao, Tangwei Teng, Xianzhong Cao, Shengpeng Wang, Senlin Hu

**Affiliations:** Center for Modern Chinese City Studies, East China Normal University, Shanghai 200062, China

**Keywords:** green innovation efficiency, knowledge diversity, threshold effect

## Abstract

Green innovation in the Yangtze River Delta is closely related to higher-quality integrated development, and knowledge diversity is crucial to the realization of regional green technology innovation and development. This study measured the green innovation efficiency of cities in the Yangtze River Delta region from 2010 to 2018 utilizing the Super-SBM model based on undesired outputs. In addition, this study used patent data to investigate regional knowledge deversity, including related variety, and unrelated variety, and to examine the spatio-temporal characteristics of green innovation efficiency and the threshold effect of knowledge diversity. The results demonstrated that related variety was positively correlated with the efficiency of urban green innovation, which was in line with extant studies. Unrelated variety was accompanied by an increase in urban science and technology investment and expansion of urban scale, and the negative effect of knowledge unrelated variety was significantly weakened. This study deepened the understanding of the mechanism of action of diversity, which is conducive to the sustainable development goals as regards the formulation of policies related to green innovation and the development of various types of cities.

## 1. Introduction

With the proposal of the United Nations Sustainable Development Goals (SDGs), the sustainable development path has received extensive attention [1,2]. The sustainable development goals aim to guide the development of the three dimensions of society, economy, and environment in an integrated manner from 2015 to 2030. Issues pertaining to “On Affordable Clean Energy” and other related requirements have brought green technology innovation to the attention of organizations. Environmental issues are serious in China as China has become the world’s largest energy consumer [3]. China is the biggest developing country; thus, taking it as a case study and exploring the relevant conclusions will help the development of global green innovation and achieving the SDGs.

There have been endless debates regarding whether specialization and diversification are the core driver of regional economic performance [4,5,6]. Since Jacobs (1961) presented big cities as natural generators of diversity, diversity has become one of the hot topics in evolutionary economic geography [7,8]. Furthermore, diversity causes various paths of economic growth, and different spillover sources have different mechanisms of action [6]. Related and unrelated variety are two kinds of diversity that differ from the degree of relevance. Related variety positively affects regional economic performance by influencing employment rate growth, whereas the role of unrelated variety is unclear [4,5]. Therefore, beyond the traditional dichotomy of specialization and diversification, the impact of related and unrelated variety merits investigation [6]. Diversification is generally based on the diversity of industries rather than knowledge [8,9]. Since Schumpeter [10] defined innovation as the reorganization of knowledge, evolutionary economic geography has focused on the importance of knowledge and innovation in regional evolution and path development.

Many countries suffer from the effects of pollution due to climate change and ongoing developmental activities [11,12]. In addition, an increasing number of countries are committed to examining methods of monitoring and supporting models of green development and innovation [13]. In the context of sustainable transformation in Evolutionary Economic Geography (EEG), it is essential to consider the particularity of green innovation, which is highly complex [14,15], and expand corresponding research assumptions, drawing on research on economic development from diversity. However, the majority of studies focus on the effect of different types of industrial agglomeration on environmental pollution and pay less attention to diversity agglomeration [16,17,18]. Only a few studies examine the stage heterogeneity of the effect of diversity on green technology innovation [19,20]. There are some controversies in the literature regarding the relationship between various types of agglomeration and green innovation, which may be due to the failure to consider the role of regional heterogeneity and endowment and the non-linear effect of diversity on green innovation.

This empirical study aimed to connect two influential theories on diversity with green innovation. This study investigated the impact of knowledge diversity on urban green innovation efficiency at the theoretical level. Moreover, this study used panel data of 41 prefecture-level cities in the Yangtze River Delta region from 2010 to 2018 to conduct an empirical analysis and measure the level of knowledge diversity and the value of urban green innovation efficiency. The spatial and temporal characteristics of urban knowledge diversity and green innovation efficiency were analyzed. Furthermore, this study used the panel threshold model to explore the non-linear impact of knowledge diversity and green innovation efficiency and investigate the impact of knowledge diversity organization models in different cities on green innovation. This study aimed to draw a differentiated development blueprint for higher-quality integration in the Yangtze River Delta.

The rest of the article is organized as follows: The second section discusses the relevant literature and provides the working hypotheses. The following section presents the data and methodology. The results of the empirical analysis, including the characteristic of spatio-temporal change, the model results, and a number of robustness tests, are presented in the penultimate section. The last section contains the main findings and conclusion.

## 2. Literature Review and Research Framework

### 2.1. Related and Unrelated Variety

In the theory of agglomeration economy, diversity was introduced by Jacobs (1961), who compared big cities to natural generators of diversity. Unlike Marshall’s focus on the impact of specialization, Jacobs considered diversity to be the main driver for increasing regional economic growth [7]. Following the integration of endogenous growth theory and economic geography in the 1990s, scholars investigated whether there is a causal relationship between regional industrial structure and innovation. In addition, cross-industry diversification [21] or industry spillover within the same sector of industry has a stronger impact on regional growth, which has become the focus of debate. Jacobs (1961) argued that the complementarity of knowledge means that effective knowledge spillover can only occur when there is the possibility of complementarity and sharing between industries [7]. Therefore, based on the above theoretical foundation, scholars have expanded and supplemented the diversity theory and proposed the concepts of related and unrelated variety [6,9,11]. Related variety goes beyond the traditional dichotomy of specialization and diversity. According to the contents of evolutionary economics, the improvement of the regional economic level relies on radical and product innovation that create new markets and jobs rather than increases in productivity [12]. Therefore, investigating the impact of related versus unrelated variety is important.

Diversity leads to various paths of economic growth, and different spillover sources have different mechanisms. There is considerable controversy in the literature regarding the role of related and unrelated diversity in the economy. Related variety is better than unrelated variety to promote localized economy, where the spillover effect comes from similar companies that produce similar products [6]. Therefore, it is expected that localized economy (related variety) will mainly affect productivity growth. Unrelated variety is based on the theory of investment restructuring [6]. Following Jacobs, unrelated variety is expected to promote radical and product innovation in different fields, as knowledge and technology of various departments are recombined, resulting in a completely new product or technology. Most studies argue that related variety has a positive impact on economic performance [8,9], whereas the impact of unrelated variety remains uncertain [8], as empirical evidence based on the theory of economic externalities suggests that diversity is not limited to knowledge diversity and involves many aspects, such as industries, products, facilities, skills, tastes, needs, and culture. In addition, there are big differences in the classification criteria and methods of related and unrelated variety. Importantly, the impact mechanism of related diversity on city economic performance is relatively complicated. Existing studies neglect the discussion of the corresponding mechanism, creating a black box effect.

### 2.2. Green Innovation

The conceptual definition of green innovation first appeared in 1996. Fussier and James [22] defined green innovation as the realization of new processes through innovation that can take into account consumers and corporate interests and environmental impacts. Subsequently, a large number of institutions and scholars represented by the Organization for Economic Co-operation and Development (OECD) have conducted studies on green innovation [23]. In a narrow sense, scholars’ definitions of green innovation focused on technology, with enterprises commonly taken as the research object. Porter et al. argued that green innovation means conforming to nature and realizing the sustainable development of enterprises through technological innovation [24] or limiting sustainable development in terms of industries, such as only focusing on how to achieve greater output with the least resource input within the scope of the industry [25]. To sum up, extant studies have vaguely defined the connotation of green innovation efficiency, mainly focusing on the regional macro- and enterprise micro-levels, with insufficient research at the meso-level. The present study argues that green innovation is a community of circular promotion formed by green development and innovation. Innovation reduces resource utilization and environmental damage in the production process through technological progress and other means, thereby promoting green development. Conversely, the requirements of green development promote technology innovation.

### 2.3. Diversity and Green Innovation

The existing research on the influencing factors of green innovation efficiency mostly begin by focusing on the urban individual, or begin by examining at a smaller level, such as enterprise organization [26]. Studies have shown that natural factors have a greater impact on urban green development [27], while economic factors such as industrial structure, human capital, energy structure, and opening to the outside world have a significant impact on green innovation efficiency [26,27]. Due to the requirements of sustainable development, diversity agglomeration and its impact on the environment is an important research area for current economics and economic geography. This type of research focuses on the impact of industrial agglomeration [28], mainly from the perspective of scale and structure. Another strand of research is rooted in recombinant innovation theory [10] and aims to explain the relationship between regional knowledge diversification and different types of innovation [29,30,31].

However, the environmental externalities of agglomeration are controversial and the effect may differ depending on the local capabilities. Copeland and Taylor [32] found that by prioritizing the invariance of governance technology, the cost of ecological pollution control in the industrial agglomeration area decreases with the increase in the return to scale, and they concluded that industrial agglomeration has a positive impact on green development. Cingano and Schivardi [17] examined micro-data from firms and revealed that industrial agglomeration can improve the efficiency of the green development of enterprises. Some studies have focused on the positive environmental externalities of technology spillovers from agglomeration [6]. Virkanen [15] demonstrated that industrial agglomeration is a major factor of environmental pollution. De Leeuw et al. [33] found that the level of industrial agglomeration was significantly correlated with air pollution. Ren [34] indicated that considering the accelerated pace of urbanization, industrial agglomeration will require land seeking to expand, resulting in overutilization and environmental pollution. Andersson [18] revealed that although industrial agglomeration can promote the efficiency of industrial output, it reduces environmental quality. The environmental externalities of agglomeration effects are controversial, and empirical research indicates that diversity and environmental pollution are non-linear, and environmental externalities are correlated with city size [30].

Scholars mostly regard green technology innovation as representative of radical innovation and focus on the relationship between regional knowledge diversity and green technology innovation, which helps analyze the multiple effect mechanism between agglomeration and green development [19]. Since Frenken [9] proposed the hypothesis of unrelated variety, there have been certain differences between related and unrelated variety in the utilization of various production factors in economic growth [31] and on the mechanism of different types of innovation [21]. Existing studies prove that the impact of diversity is heterogeneous from the aspects of the green technology innovation life cycle [19], whereas the mechanism of unrelated diversity on regional development and technological innovation remains unclear [31].

Both strands of literature show that the relationship between diversity and green innovation is complex and unclear. Research on the relationship between knowledge diversity and green innovation ignores cities’ capabilities. A previous study identified scale, industry structure, and technology as the main factors affecting the environmental quality [35]. In big cities with high scales, the agglomeration of similar industries is conducive to the sharing of the labor market and reducing production risks and costs, thereby improving efficiency and reducing pollution levels [21]; however, with the expansion of the scale of agglomeration, the crowding effect of capital, labor, and technology promotes industrial growth, resulting in increased pollution emissions [36]. A high proportion of secondary industries in cities leads to the entry of high-polluting industries and causes harm to the environment; however, high-polluting industries force regions to strengthen management and control. Diversified agglomeration makes it easier to achieve internal material exchange between related industries, and recycling of materials between enterprises facilitates pollution reduction and helps improve the efficiency of urban green innovation [37,38]. In cities with different technological levels, diversity has heterogeneity in the mechanism of green innovation efficiency. In EEG, technological linkages among industries with related variety are an important condition for technological spillovers. Compared with the agglomeration of unrelated industries, industries with cognitive relatedness are easier to cooperate, which promotes technology spillover, improves the level of innovation [39,40], and reduces the emission intensity of production waste, whereas related variety is more beneficial for radical innovation represented by green technology innovation [19,31]. In addition, technology misuse poses a risk of contamination [41]. Studies provide theoretical support for the existence of a threshold effect on the role of diversity; however, empirical research is lacking.

### 2.4. Research Framework

**Figure 1:**
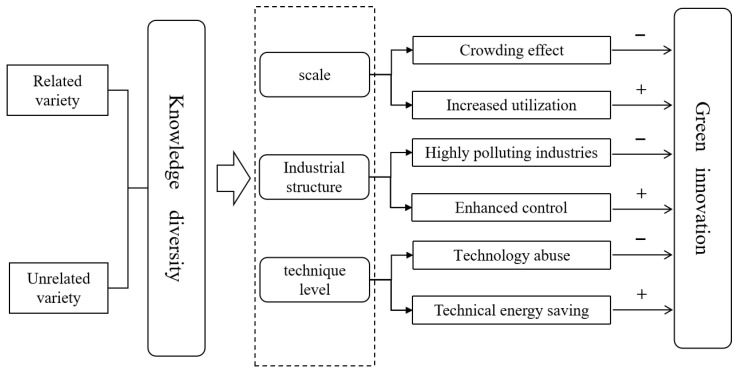
Research framework of the effect of knowledge diversity on green innovation.

Knowledge diversity has a decisive influence on promoting green innovation which has two attributes: green and innovative development. Knowledge diversity is a type of agglomeration, and in addition to the multiple effects of agglomeration commonality, related variety, and unrelated variety, it has unique technical effects on various types of innovation and at different scale levels. Therefore, to improve the efficiency of green innovation, the impact of agglomeration externalities on productivity improvement and its environmental effects and the knowledge attribute of innovation should be considered. The research framework of the effect of knowledge diversity on green innovation was constructed from the externalities of agglomeration form, considering city scale, the industrial structure and technical effects (Figure 1), which will act on regional green development along different paths [23]. Existing studies have confirmed that, in the process of industrial agglomeration, there may be differences between positive and negative externalities [31]; thus, we believe that, due to the multi-attribute characteristics of green innovation, the effect mechanism of diversity on green innovation is complex rather than a simple linear relationship, and further analysis of the threshold effect is required. Considering that city scale, industrial structure, and the technique level, as the main factors affecting the environmental quality, significantly affect knowledge diversity’s decisive influence on promoting green innovation [39,40], we take scale, industrial structural, and technique level as threshold variables.

## 3. Materials and Methods

### 3.1. Study Area and Data Sources

According to the regional typicality and latest policy, this study selected the Yangtze River Delta with 41 cities as case sites based on the Outline of the Yangtze River Delta Regional Integrated Development Plan issued in December 2019. The Yangtze River Delta is a highland for China’s green innovation and development and plays a leading role nationwide in terms of policy and scientific and technological research. The region’s economy has developed rapidly, and the regional innovation level has improved significantly. In 2019, the Yangtze River Delta region, which accounts for less than 4% of the country’s land area, contained 16% of the population, accounting for nearly 1/4 of the total economic volume. It generated approximately 1/4 of the scientific research force and approximately 1/3 of the effective invention patents. The improvement of the ecological environment has been remarkable. In 2019, the average concentration of PM 2.5 in 41 cities was 41 micrograms per cubic meter, and the ratio of good days was 76.5%. At the policy level, the Yangtze River Delta has recently vigorously promoted the construction of ecological and green integrated development demonstration zones and established the Yangtze River Delta Green Development Professional Committee, which is committed to realizing the organic unity of the green economy and sustainable development and exploring new paths for co-construction and sharing across administrative regions, ecological civilization, and economic and social development.

Based on data availability and stationarity, nine years from 2010 to 2018 were selected as the study period. Three types of research data were included. First, the statistical yearbook data were mainly obtained from the China Urban Statistical Yearbook, China Environmental Statistical Yearbook, China Industrial Statistical Yearbook, China Energy Statistical Yearbook, provincial and municipal statistical yearbooks, and government bulletins. Second, patent data were based on a patent database (www.incopat.com, accessed on 2 February 2022), which was constructed by cleaning data and merging the year and city by the filing date. Third, geographic data were obtained from the vector data of prefecture-level cities in China provided by the Chinese Academy of Sciences’ resource and environmental data cloud platform. The missing values are mainly related to new products, among which the sales revenue data of new products in cities in Zhejiang Province are supplemented by the output value data of new products. In addition, there are small amounts of data missing in the yearbooks, which are supplemented by the average value of adjacent years. In addition, the full sample data were processed to eliminate outliers and supplemented by interpolation. The statistical caliber of certain data changed in 2019. The statistical caliber of the China Energy Statistical Yearbook changed in 2019. In addition, the statistical caliber of R&D-related indicators changed. According to the Statistical Specifications for Investment in R&D and Experimental Development (Trial Implementation), R&D expenditures for research and experimental development are divided into internal expenditures and external expenditures according to the main body of the expenditure. Therefore, our study period ends in 2018 to ensure data stationarity.

### 3.2. Green Innovation Efficiency Measurement

#### 3.2.1. Green Innovation Efficiency Index Construction

Green innovation efficiency considers both input and output. Green innovation efficiency is more accurate than development level [42,43]. Considering the difference between technological innovation levels of China and European countries and the relatively small amount of green technological innovation in China, this study used a composite concept to conduct research combined with research questions. Green innovation efficiency was used, with as little input as possible to obtain greater innovation output. This study selected indicators from the perspective of input and output based on ecological economics and measured the efficiency of green innovation in 41 cities in the Yangtze River Delta region. Based on the concept of green innovation, five secondary indicators and eight tertiary indicators were constructed (Table 1). Furthermore, based on the emphasis on labor and capital in the Cobb–Douglas production function and considering the definition of innovation, three indicators of investment (R&D, energy, and capital) were selected. Moreover, considering the double spillover effects of green innovation in knowledge and environmental protection spillovers, the output indicators were considered from both innovation and environment aspects.

#### 3.2.2. Super-SBM

Green innovation efficiency can be used to measure the city’s green innovation level from the perspective of input and output and reflect the green innovation development status of the measurement unit [47,48,49]. The traditional DEA model has certain limitations. It is greatly affected by environmental factors and random disturbances. It cannot effectively distinguish decision-making units located on the same frontier, and ignores the influence of slack variables and undesired outputs. Compared with the traditional data envelopment analysis (DEA) model, the undesired-based super-slacks-based measure (SBM) model takes the undesired output into consideration and is suitable for considering environmental effects [50]. This model distinguishes multiple units with the green innovation efficiency value of each city for the year, and the result of the super-SBM is more comparable than the result of the SBM. The calculation formula is as follows:

(1) $$\rho =\min\frac{1-\frac{1}{m}\sum_{i=1}^{m}\frac{p_{i}^{-}}{x_{i_{o}}}}{1+\frac{1}{p_{1}+p_{2}}\left[\sum_{r=1}^{p_{1}}y_{r_{0}}^{g}{}^{p_{r}^{g}}+\sum_{r=1}^{p_{2}}y_{r_{0}}^{b}{}^{p_{r}^{b}}\right]}$$

(2) $$s.t.\left\{\begin{array}{c} x_{0}=X\beta +p^{-}\\ y_{0}^{g}=Y^{g}\beta -p^{g}\\ y_{0}^{b}=Y^{b}\beta +p^{b}\\ p^{-}\geq 0,\;p^{g}\geq 0,\;P^{b}\geq 0,\;\beta \geq 0 \end{array}\right.$$

The three feature vectors *x*, $\;y^{g},\;y^{b}$ represent input and expected and undesired output, respectively. *X*, $Y^{g},\;Y^{b}$ refer to three matrixes with a dimensional of m plus *n*, $X=\left[x_{1},\;x_{2},\;\ldots ,\;x_{n}\right]\in R^{m\times n}$. $p^{-}\;\in \;R^{m},\;p^{g}\;\in \;R^{p_{2}}$ denote the excess using the vector of the input and undesired output. The evaluation model of the decision-making unit is shown in Formula (1). The function strictly decreases. When *ρ* = 1, the decision-making unit is at the best production frontier.

Using Formula (1) for the calculation, the result is prone to give the efficiency of multiple decision-making units as 1. To distinguish this type of decision-making unit by analogy, the decision-making unit is processed as follows:

(3) $$P=\min\frac{\frac{1}{m}\sum_{i=1}^{m}\frac{p_{i}^{-}}{x_{i_{o}}}}{\frac{1}{p_{1}\;+\;p_{2}}\left[\sum_{r=1}^{p_{1}}y_{r_{0}}^{g}{}^{p_{r}^{g}}+\sum_{r=1}^{p_{2}}y_{r_{0}}^{b}{}^{p_{r}^{b}}\right]}$$

(4) $$s.t.\left\{\begin{array}{c} \bar{x}=\sum_{j=1,\neq 0}^{n}x_{j}\beta_{j}\\ \bar{y^{g}}=\sum_{j=1,\neq 0}^{n}y_{j}^{g}\beta_{j}\\ \bar{y^{b}}=\sum_{j=1,\neq 0}^{n}y_{j}^{b}\beta_{j}\\ \bar{x}\geq x_{0},\;\bar{y^{g}}\geq y_{0}^{g},\;\bar{y^{b}}\geq 0,\;\beta \geq 0 \end{array}\right.$$

### 3.3. Regional Knowledge Diversification

Assuming that relatedness between patents increases together with the number of International Patent Classification (IPC) digits in which two patents are the same, we calculated related (*RV*) and unrelated (*UV*) variety using the IPC code at a different level of disaggregation (Barbieri et al. [19]). First, *UV* was measured using the entropy of the patent family distribution over IPC three-digit classes (i.e., *UV*).

(5) $$UV_{it}=\sum_{k=1}^{n}pf_{k,it}\ln(\frac{1}{pf_{k,it}})$$

where *pf_k_* is the share of patent families in a technological section *k* = [1 … *N*] at the IPC three-digit level, with at least one inventor located in city *i* at time *t*.

Given the decomposition theorem developed by Theil [51], *RV* is the difference between the entropy measure calculated at the four-digit and three-digit levels (i.e., *UV*):

(6) $$RV_{it}=\sum_{l=1}^{m}pf_{l,it}\ln(\frac{1}{pf_{l,it}})-UV_{it}$$

where *pf_l_* is the share of technological section k’s (*k* = [1 … *N*]) patent families at the IPC four-digit level, with at least one inventor located in state *i* at time *t*.

### 3.4. Econometric and Estimation Methods

The stochastic impacts of regression on the population, affluence, and technology (STIRPAT) are widely used in the field of environmental economics [52]. York et al. (1994) improved the IPAT model and proposed the STIRPAT model, which allows the specific decomposition of certain influencing factors of technology, population, and wealth and makes it possible to specifically examine the impact of a certain factor on the environment [52]. Based on the knowledge production function, this study expanded the technical level and built a measurement model of regional knowledge diversity and its relationship with urban green innovation efficiency. The formula is as follows:

(7) $$\begin{array}{l} GIE_{it}=a_{i}+\beta_{1}RV_{it}+\beta_{2}\ln er_{it}{}^{{}^{}}+\beta_{3}\ln fdi_{it}+\beta_{4}is_{it}+\beta_{5}\ln rgdp_{it}+\beta_{6}\ln te_{it}+\varepsilon_{it} \end{array}$$

(8) $$\begin{array}{l} GIE_{it}=a_{i}+\beta_{1}UV_{it}+\beta_{2}\ln er_{it}{}^{{}^{}}+\beta_{3}\ln fdi_{it}+\beta_{4}is_{it}+\beta_{5}\ln rgdp_{it}+\beta_{6}\ln te_{it}+\varepsilon_{it} \end{array}$$

where *GIE* is the dependent variable, indicating the efficiency level of urban green innovation; *RV*, *UV*, ln*er*, *fdi*, *is*, *rgdp*, and *te* are unrelated diversity, environmental regulation, foreign investment, industrial structure, urban economic scale, and technology expenditure, respectively; and *β*_1_, *β*_2_, *β*_3_, *β*_4_, *β*_5_, and *β*_6_ are their coefficients, respectively. The descriptive statistics of variables are shown in Table 2.

The threshold model plays an important role in exploring the relationship between variables and the law of mutation among variables [53]. This study drew on the Hansen threshold model [54] and expanded the model based on the knowledge production function. We quantitatively analyzed the threshold characteristics of the relationship between urban knowledge diversity and green innovation in terms of scale, structure, and technology and empirically obtained the specific conditions for knowledge diversity to act on green innovation. The formulas are as follows:

(9) $$GIE_{it}=a_{i}+\beta_{1}UV_{it}*I(IS_{it}\leq \gamma )+\beta_{1}UV_{it}*I(IS_{it}>\gamma )+\beta_{3}H+\varepsilon_{it}$$

(10) $$GIE_{it}=a_{i}+\beta_{1}UV_{it}*I(\ln rgdp_{it}\leq \gamma )+\beta_{1}UV_{it}*I(\ln rgdp_{it}>\gamma )+\beta_{3}H+\varepsilon_{it}$$

(11) $$GIE_{it}=a_{i}+\beta_{1}UV_{it}*I(\ln te_{it}\leq \gamma )+\beta_{1}UV_{it}*I(\ln te_{it}>\gamma )+\beta_{3}H+\varepsilon_{it}$$

(12) $$GIE_{it}=a_{i}+\beta_{1}RV_{it}*I(IS_{it}\leq \gamma )+\beta_{1}RV_{it}*I(IS_{it}>\gamma )+\beta_{3}H+\varepsilon_{it}$$

(13) $$GIE_{it}=a_{i}+\beta_{1}RV_{it}*I(\ln rgdp_{it}\leq \gamma )+\beta_{1}RV_{it}*I(\ln rgdp_{it}>\gamma )+\beta_{3}H+\varepsilon_{it}$$

(14) $$GIE_{it}=a_{i}+\beta_{1}RV_{it}*I(\ln te_{it}\leq \gamma )+\beta_{1}RV_{it}*I(\ln te_{it}>\gamma )+\beta_{3}H+\varepsilon_{it}$$

where *GIE* represents green innovation efficiency; *i* and *t* represent prefecture-level cities and years, respectively; *is* is the proportion of secondary production value, which is used to characterize the industrial structure; *te* is science and technology expenditure; and *rgdp* is the per capita GDP of the corresponding city. *I* represents the threshold variable, *γ* is the threshold value, and *H* is used to represent the control variable. Due to space limitations, only the single-threshold model is listed, and the double- and triple-threshold models are expanded based on this.

## 4. Results

### 4.1. Spatio-Temporal Change of Related Variety, Unrelated Variety, and Green Innovation Efficiency

#### 4.1.1. Temporal Variation of Related Variety, Unrelated Variety, and Green Innovation Efficiency

**Figure 2:**
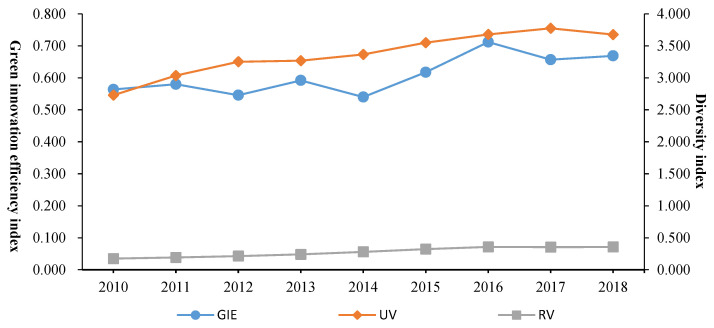
Time series characteristics of green innovation efficiency and related variety and unrelated variety in the Yangtze River Delta from 2010 to 2018.

Figure 2 shows the changes in green innovation efficiency and knowledge diversity in the Yangtze River Delta region from 2010 to 2018. The green innovation efficiency in the Yangtze River Delta region shows a fluctuating growth trend. The growth trends of related variety and unrelated variety were similar, showing a steady and slow growth trend, which differed from the fluctuation trend of green innovation efficiency. Before 2014, green innovation development did not receive attention, and economic development was the main task at this stage. In 2014, with the formal proposal of the green development strategy of the Yangtze River Economic Belt, the level of green innovation in the Yangtze River Delta region began to improve significantly, but in 2016, there was a relatively obvious change point. Influenced by the implementation of the supply-side reform, the Yangtze River Delta region accelerated the industrial structural reform this year. The related variety index was significantly lower than the unrelated variety index, and the related variety fluctuated less than the unrelated variety. Green innovation efficiency showed a fluctuating downward trend from 2011 to 2014. In 2014, the green innovation efficiency value was 0.54, which was the lowest level in the research period. In 2013, the green innovation efficiency in the Yangtze River Delta region showed a fluctuating growth. The development of green innovation in the Yangtze River Delta region was clearly affected by the lag of the financial crisis in 2008. In the early stage of the study period, the production of some enterprises in the region was stagnant, the investment in scientific research was affected, and the efficiency of green innovation dropped significantly. During the same period, the efficiency of green innovation in the Guangdong-Hong Kong-Macao Greater Bay Area showed a similar recession trend [55].

#### 4.1.2. Spatial Variation of Related Variety, Unrelated Variety, and Green Innovation Efficiency

**Figure 3:**
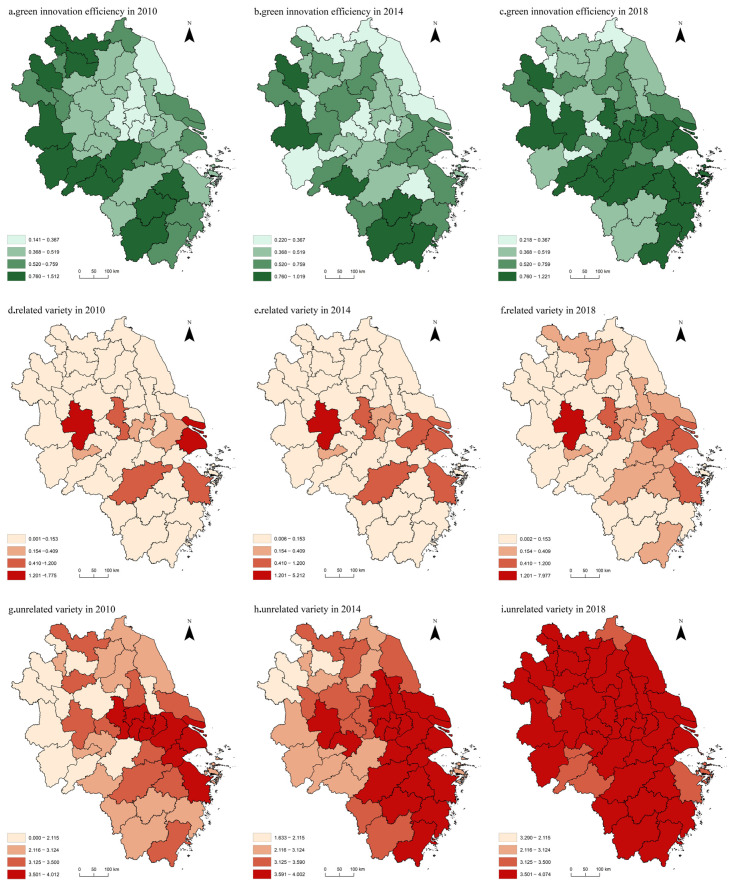
Spatial distribution of green innovation efficiency and related variety and unrelated variety in the Yangtze River Delta from 2010 to 2018.

To obtain the spatial distribution characteristics and differences of green innovation efficiency and knowledge diversity in the Yangtze River Delta region, three node years (2010, 2014, and 2018) were selected. ArcGIS was used to select the natural break nodes in the middle years as break points for reclassification (Figure 3).

During the study period, the center of green innovation in the Yangtze River Delta region moved eastward. The spatial pattern of related diversity was relatively stable, showing a small-scale flaky dispersion. The spatial variation characteristics of unrelated variety differed from that of related variety, showing the development from flaky agglomeration to multi-core, and the dispersion trend was clearer. From the perspective of relative change trends, the green innovation efficiency in the Yangtze River Delta region was greatly affected by the urban natural background in 2010, i.e., the areas with higher green innovation efficiency were mainly distributed in southern Anhui, western Anhui, and southern Zhejiang with superior natural conditions. During the research period, the dependence of green innovation efficiency on scientific and technological innovation capability gradually increased [26]. In 2018, the regions with high green innovation efficiency were Shanghai, Nanjing, Hangzhou, Hefei, and other regional core cities with a high technological level. The spatial pattern of related variety changed little, forming a relatively significant polarization pattern of core cities. In addition, there is a relatively clear diffusion effect around Shanghai, which is related to the construction of Shanghai Hongqiao International Hub, comprising “one core and two belts”. The policy made the area closer and more integrated.

### 4.2. The Influence of Related Variety and Unrelated Variety on Green Innovation

#### 4.2.1. Base Model

To ensure scientific accuracy, this study first conducted the LR test. The results rejected the hypothesis of the mixed model, and the Hausman test result rejected the null hypothesis regarding the support random effect model (Prob > chi2 = 0.0000); therefore, the panel with double fixed individual time was selected [56]. This study regressed the data from 2010 to 2018 during the study period and discussed how relevant and unrelated diversity affected the efficiency of urban green innovation in the Yangtze River Delta region (Table 3).

Knowledge-related variety in the Yangtze River Delta region had a significant positive impact on urban green innovation efficiency, whereas unrelated variety had a negative impact. As shown in Model (1) in Table 3, the coefficient of related variety was 0.059, which was significant at the 5% level, and the coefficient of related variety was the largest, which indicated that related variety was an important source of urban green innovation efficiency improvement. Related variety agglomeration made it easier to achieve intrinsic material exchange between related industries, and the recycling of materials between enterprises helped achieve pollution reduction and improve the efficiency of urban green innovation. This was in line with extant studies that indicate that relevant knowledge reorganization can promote corresponding green technology innovation [19,31]. In addition, as shown in Model (2), the coefficient of unrelated variety was −0.16, which was significant at the 1% level. With the expansion of the scale of agglomeration, the scale effect of capital, labor, and technology promoted industrial growth, resulting in an increase in pollution emissions, and unrelated variety reduced resource utilization due to the lack of technological connections. This study indicated that the technical effects of unrelated diversity were complex, which was consistent with existing research that argues that unrelated diversity has different mechanisms for different types of innovation [19,31]. Given the multiple effects of knowledge diversity, there may be a non-linear relationship between knowledge diversity and urban green innovation efficiency, and the related mechanisms require further empirical research concerning the threshold effect of knowledge diversity while considering different types of cities’ local capabilities.

In terms of control variables, per capita GDP and technology investment were both positive and significant, which echoed the conclusion at the enterprise level that large companies play a leading role in the process of green innovation [57]. Technological investment positively affected green innovation efficiency by improving technological efficiency. The industrial structure showed a significant negative trend, which confirmed the general conclusion of industrial structure and green innovation, i.e., industrial manufacturing inevitably caused environmental pollution. This further demonstrated that the research framework of “scale-structure-technology” has a strong explanatory power for the mechanism of green innovation efficiency. The outward dependence of development was significantly weakened, and the region passed the “pollution shelter effect” phase during the study period.

#### 4.2.2. Threshold Effect

Three explanatory variables (ln*rgdp*, *is*, and ln*te*) were selected from the three levels of city scale, industry structure, and technology level. Based on the Hansen threshold effect test [54], the threshold effect test results for the three variables revealed the impact of urban knowledge diversity on the efficiency of urban green innovation. In the related variety mechanism (Table 4), there was a single-threshold effect at the scale level, and the corresponding threshold value was 11.62, which passed the single-threshold test at the 1% level but failed the threshold effect test at the structural and technical levels. The mechanism of unrelated variety was more complicated than that of related variety (Table 5). The scale and technical levels passed the single-threshold effect test, and the corresponding threshold values were 11.62 and 13.30, which passed the threshold test at the significant levels of 1% and 10%, respectively. There was no threshold effect at the structural level.

Related and unrelated variety had a single-threshold effect on green innovation efficiency at the scale level, whereas non-related diversity had a single-threshold effect at the technical level. Different urban economic scales and technological investment levels led to regional differences in the growth of green total factor innovation efficiency, which echoed previous studies [58]. However, related and unrelated variety had different degrees of technology dependence on green innovation, and unrelated diversity had a higher degree of technology dependence [22], which echoed extant research on the impact of industrial diversification on green total factor productivity. The role was affected by the technology mediation effect [59]. In terms of industrial structure, there was no threshold for related and unrelated variety, as the Yangtze River Delta and China were undergoing economic structure optimization and adjustment. Changes were small during the research period, and they did not enter the high-level stage; therefore, they did not show stage heterogeneity.

In addition, this study examined the effect of knowledge diversity on the efficiency of urban green innovation when the scale, structure, and technology were in different threshold ranges. The regression results for related diversity on urban green innovation efficiency are shown in Table 4. Related variety had a threshold effect on green innovation efficiency only when scale was used as the threshold variable. The effect was positive, and the influence coefficient continued to increase. When lnrgdp was less than the threshold value of 11.62, the influence coefficient of knowledge-related diversity on urban green innovation efficiency was 0.069, which was significant at the level of 1% and had a positive promoting effect. The influence coefficient of urban green innovation increased to 0.504, and the significance level remained high. This demonstrated that with the expansion of urban economic scale, the Yangtze River Delta region was in a period in which the agglomeration of knowledge-related diversity had stronger positive than negative externalities considering the cost of technological innovation [60], thereby improving the efficiency of green innovation.

The regression results for unralated diversity on urban green innovation efficiency are shown in Table 5. Unrelated variety had a threshold effect on green innovation efficiency when structural and technical variables were used as threshold variables. When the scale variable was used as the threshold variable, all variables exhibited a negative effect. With the increase in the urban economic scale, the negative effect was weakened, and the significance decreased. When the urban economic scale was used as the threshold variable, and lnrgdp was less than the threshold value of 11.62, the influence coefficient of knowledge unrelated diversity on the efficiency of urban green innovation was 0.112, which was negative and significant at the 1% level. When the scale increased and the threshold value was crossed, the negative impact of knowledge diversity on urban green innovation was significantly weakened, and the significance test was not passed. This indicated that with the expansion of urban economic scale, the problem of environmental pollution became prominent, and the demand for improving the efficiency of green innovation changed from the increase in the number of patents and output value of new products to reducing the pressure on per capita resources and environment [61]. Unrelated variety offers the possibility of generating new product chains and sharing resources [62].

When technology input was used as a threshold variable, unrelated variety had a negative correlation with urban green innovation efficiency. With the increase in urban science and technology investment, the negative effect of knowledge unrelated variety was significantly weakened, while its significance remained high. When the investment in science and technology was lower than the threshold value, the coefficient of knowledge unrelated variety was −0.135. After crossing the threshold value, the influence coefficient became −0.081, which remained significant at more than 5% level. Unrelated variety was more sensitive to technological investment than related variety. Unrelated variety had different mechanisms for different types of innovations and mostly acted on radical innovations, which often require greater capital investment. With the increase in urban technological investment, the effect of unrelated variety on innovation was significantly enhanced [19], and its positive technological externalities gradually offset the negative externalities of unrelated diversity agglomeration.

To sum up, knowledge-related diversity had a large spatial and temporal heterogeneity in green innovation efficiency based on the endowments of different cities and showed significant threshold characteristics. When urban development entered a new stage, i.e., after the threshold variable crossed the threshold value, knowledge diversity affected the evolution of urban green innovation efficiency in a new way. The evolution of green innovation efficiency in the Yangtze River Delta region was characterized by the circular accumulation and joint promotion of many factors, such as urban economic scale, urban science and technology investment, and urban industrial structure.

Based on the threshold value identification, the number of prefectures and cities in the Yangtze River Delta region located in different threshold value ranges was counted, and their proportions were calculated to explore their evolution trends. The results in Table 6 demonstrated that most cities in the Yangtze River Delta region were below the threshold level in terms of scale and technology, and related variety remained in the stage of weak positive environmental externality, whereas unrelated variety was in the negative environmental externality due to weak technical level. At this stage, the role of knowledge diversity in improving the efficiency of urban green innovation required enhancement. Therefore, it can be considered to enhance the promotion effect of regional knowledge diversity on the improvement of urban green innovation efficiency by accelerating the improvement of urban economic scale and scientific and technological investment.

## 5. Discussion

Based on the analysis of the threshold effect of knowledge diversity on green innovation, this study further enriched the discussion of agglomeration and green development in the existing literature and explored evolutionary and environmental economic geography. The conclusions echo the view in the existing literature, i.e., diversity has stage heterogeneity in green technology innovation [19]. This demonstrated that the mechanism of action was closely related to regional capabilities [63]. Furthermore, the results differed from the relevant conclusions in existing studies that ignore the regional capabilities and argue that there is a simple linear relationship between agglomeration and green development [64]. This study makes an effective attempt to open the “black box” of green innovation and development, initially reveals that the connotation of green innovation development may differ in different circumstances, and provides theoretical support for the differentiated development of various types of cities. Relevant research conclusions are helpful for the green development of cities in the Yangtze River Delta region. The conclusions also provide insights that can help different types of cities determine their own green innovation efficiency improvement paths in order to alleviate regional development imbalances and break through regional green development bottlenecks. These are not only applicable to the Yangtze River Delta, but may also have implications for other similar regions in the world, and can ultimately contribute to the achievement of SDGs. Future studies should start from the development and evolution of green innovation and further explore the subject and the path of knowledge diversity. This line of research is of great significance for exploring the heterogeneity of green development in different regions.

## 6. Conclusions

Based on the perspective of urban green development and innovative development, this study examined the temporal and spatial evolution characteristics of knowledge diversity and urban green innovation efficiency in the Yangtze River Delta region from 2010 to 2018 and the threshold effect of knowledge diversity, which extended research about green efficiency promotion strategies of different types of cities to achieve SDGs. The following conclusions could be drawn.

First, the spatial and temporal distribution and change characteristics of urban green innovation efficiency and knowledge diversity differed. The green innovation efficiency in the Yangtze River Delta region showed a fluctuating growth trend. Related and unrelated variety in the Yangtze River Delta region had similar growth trends, indicating a steady and slow growth trend, which differed from the fluctuation trend of green innovation efficiency. The center of green innovation in the Yangtze River Delta region moved eastward. Related variety developed from multi-core to sheet-like agglomeration, whereas unrelated variety exhibited the opposite trend, showing development from sheet-like agglomeration to multi-core development and clearer scattering.

Second, related variety had a threshold effect on green innovation efficiency only when city scale was used as the threshold variable, all variables had a positive effect, and the influence coefficient increased. When technology investment and industrial structure were used as threshold variables, the correlation between diversity and green innovation efficiency was linear, which failed the threshold test.

Third, unrelated variety had a threshold effect on green innovation efficiency when the variables of industry structure and technology were used as threshold variables. When the scale variable was used as the threshold variable, all variables exhibited a negative effect. With the increase in the urban economic scale, the negative effect weakened, and the significance decreased. When technology input was used as a threshold variable, unrelated variety had a negative correlation with urban green innovation efficiency. With the increase in urban science and technology investment, the negative effect of knowledge unrelated variety significantly weakened; however, its significance remained at a relatively high level.

Finally, most cities in the Yangtze River Delta region were below the threshold level in terms of scale and technology; related variety remained in the stage of weak positive environmental externalities, whereas unrelated variety was in the stage of relatively negative environmental externalities due to its weak technical level at the strong stage.

## Figures and Tables

**Table 1:** Evaluation index system of green innovation efficiency in the Yangtze River Delta.

First-Level Indicator	Second-Level Indicator	Third-Level Indicator	
Input	R&D investment	Number of people engaged in R&D activities in industrial enterprises	Li et al. [44] Kneller et al. [45]
	Energy input	Industrial comprehensive energy consumption	Li et al. [44]
	Capital investment	Total industrial fixed asset investment	Zhou et al. [27]
Output	Expected output	Number of patents	Kneller et al. [45]
		New products	
	Unexpected output	Waste gas	Managi and Kaneko [46]
		Sewage	
		General industrial solid-waste emissions	

**Table 2:** Descriptive statistics of variables.

Theme	Variable	Calculation Method	Mean	Std. Dev.	Min	Max	Obs.
Dependent variable	Green innovation efficiency (*GIE*)	Calculated by Super-SBM	0.614	0.254	0.141	1.512	369
Explanatory variable	Unrelated variety (*UV*)	Count of patents’ entropy in a city	3.378	0.653	0	4.136	369
	Related variety (*RV*)	Count of patents’ entropy in a city	0.277	0.904	0	8.233	369
Control variables	Environmental regulation (ln*er*)	Environmental protection investment in GDP (%)	0.544	0.55	0.011	3.859	369
	Openness (ln*fdi*)	Foreign direct investment	11.299	1.267	8.181	14.431	369
	Industrial structure (*is*)	Output value of secondary industry in GDP (%)	48.719	7.962	29.78	74.735	369
	Economy size (ln*rgdp*)	GDP per capita	10.891	0.607	9.162	12.048	369
	Technology level (ln*te*)	Tech spending	11.475	1.226	8.176	15.266	369

**Table 3:** Empirical results of knowledge diversity in the Yangtze River Delta region on the efficiency of urban green innovation from 2010 to 2018.

Variables	(1)	(2)	(3)	(4)
Related Variety (*RV*)	0.059 ** (0.023)		0.051 ** (0.025)	
Unrelated Variety (*UV*)		−0.160 *** (0.030)		−0.216 *** (0.039)
Economy size (ln*rgdp*)	0.124 * (0.066)	0.255 *** (0.069)	0.224 ** (0.088)	0.416 *** (0.090)
Industrial structure (*is*)	−0.008 *** (0.003)	−0.008 *** (0.003)	−0.009 ** (0.003)	−0.007 ** (0.003)
Technology level (ln*te*)	−0.054 * (0.030)	−0.008 (0.030)	−0.055 (0.044)	−0.002 (0.041)
Openness (ln*fdi*)	−0.002 (0.030)	0.044 (0.030)	0.003 (0.038)	0.060 (0.037)
Environmental regulation (*er*)	−0.006 (0.024)	−0.006 (0.023)	−0.053 (0.035)	−0.046 (0.033)
_cons	0.271 (0.606)	−1.610 * (0.678)	−0.848 (0.888)	−3.589 *** (0.953)
Time fixed	Y	Y	Y	Y
City fixed	Y	Y	Y	Y
N	369	369	243	243

Notes: Standard errors in parentheses; * *p* < 0.1, ** *p* < 0.05, *** *p* < 0.01.

**Table 4:** Regression results of the threshold effect of various variables for related variety of knowledge on the efficiency of urban green innovation.

Variables	*RV*-1	*RV*-2	ln*er*	ln*fdi*	ln*rgdp*	*Is*	ln*te*	*Cons*
Scale	0.069 *** (0.022)	0.504 *** (0.088)	−0.001 (0.023)	0.020 (0.029)	0.059 (0.065)	−0.006 *** (0.002)	−0.055 (0.029)	0.656 (0.587)
Structure	-	-	-	-	-	-	-	-
Technique	-	-	-	-	-	-	-	-

Notes: Standard errors in parentheses; *** *p* < 0.01.

**Table 5:** Regression results of the threshold effect of unrelated variety of knowledge on the variables of urban green innovation efficiency.

Variables	*UV*-1	*UV*-2	*UV*-3	ln*er*	ln*fdi*	ln*rgdp*	*Is*	ln*te*	*Cons*
Scale	−0.112 *** (0.031)	−0.052 (0.036)		0.010 (0.023)	0.062 (0.029)	0.113 (0.071)	−0.007 *** (0.002)	−0.013 (0.029)	−0.471 (0.686)
Structure	-	-	-	-	-	-	-	-	-
Technique	−0.135 *** (0.031)	−0.081 ** (0.039)	-	−0.005 (0.023)	0.045 (0.030)	0.245 *** (0.068)	−0.008 ** (0.002)	−0.040 (0.031)	−1.304 * (0.675)

Notes: Standard errors in parentheses; * *p* < 0.1, ** *p* < 0.05, *** *p* < 0.01.

**Table 6:** Proportion of cities with less than threshold estimates in the Yangtze River Delta region.

Year	ln*rgdp* < 11.62 (%)	ln*te* < 13.3 (%)
2010	100	97.6
2012	90.2	95.1
2014	95.1	95.1
2016	80.5	90.2
2018	73.2	82.9

## Data Availability

Data sharing not applicable.
